# Adjunctive treatment of symmetrical peripheral gangrene with phenolamine wet compress

**DOI:** 10.1097/MD.0000000000044099

**Published:** 2025-08-22

**Authors:** Yichao Zhu, Tao Zhou, Haohao Qian, Hongyang Xu

**Affiliations:** a Department of Critical Care Medicine, the Affiliated Wuxi People’s Hospital of Nanjing Medical University, Wuxi People’s Hospital, Wuxi Medical Center, Nanjing Medical University, Nanjing, China.

**Keywords:** phentolamine, septic shock, symmetrical peripheral gangrene

## Abstract

**Rationale:**

Symmetrical peripheral gangrene (SPG) is a rare and life-threatening condition characterized by symmetrical distal ischemic damage that can progress to gangrene at 2 or more anatomical sites, without involvement of a large vessel obstruction or vasculitis. We report the case of an older adult female who developed septic shock and disseminated intravascular coagulation (DIC) postoperatively, complicated by the onset of SPG.

**Patient concerns:**

A 66-year-old female was transferred to the intensive care unit for postoperative management. She subsequently developed septic shock and DIC. On the 10th day of admission, acral necrosis was observed in both feet.

**Diagnoses:**

Septic shock complicated by DIC and SPG.

**Interventions:**

Upon intensive care unit admission, the patient received ongoing organ support and anti-infection therapy. Phentolamine was applied via wet compresses to both feet.

**Outcomes:**

Due to anastomotic leakage and multiple organ failure, the patient’s family chose to discharge her from the hospital.

**Lessons:**

In cases of SPG, prevention is more important than treatment. Early correction of underlying conditions and identification of risk factors can improve patient outcomes.

## 1. Introduction

Symmetrical peripheral gangrene (SPG) is a rare and life-threatening complication most frequently seen in patients with heart failure, septic shock, or disseminated intravascular coagulation (DIC). It can affect individuals of any age or sex. Once diagnosed, SPG has a mortality rate of 40% to 90% and an amputation rate of up to 50%.^[1]^ Clinically, SPG is characterized by symmetrical distal ischemic injury leading to gangrene in 2 or more locations, not associated with large-vessel occlusion or vasculitis.^[2]^ Due to its complex etiology, no standardized global guideline currently exists for the diagnosis and treatment of SPG. Phentolamine has been reported in several cases to be effective in treating skin necrosis caused by the extravasation of vasoactive drugs.^[3]^ However, its use in managing SPG has rarely been documented. Here, we report the case of an older adult female who developed postoperative septic shock and DIC, complicated by concurrent SPG. The patient was treated with phentolamine wet compresses, resulting in a favorable outcome.

## 2. Case report

**Figure 1.**
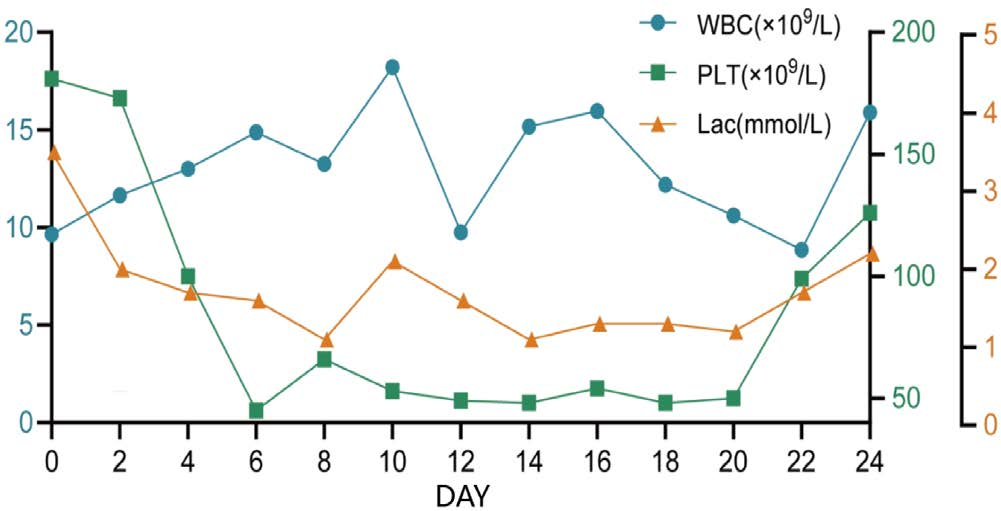
Changes in laboratory indicators during the patient’s hospitalization in the ICU. ICU = intensive care unit. PLT = platelets, WBC = white blood cells

**Figure 2.**
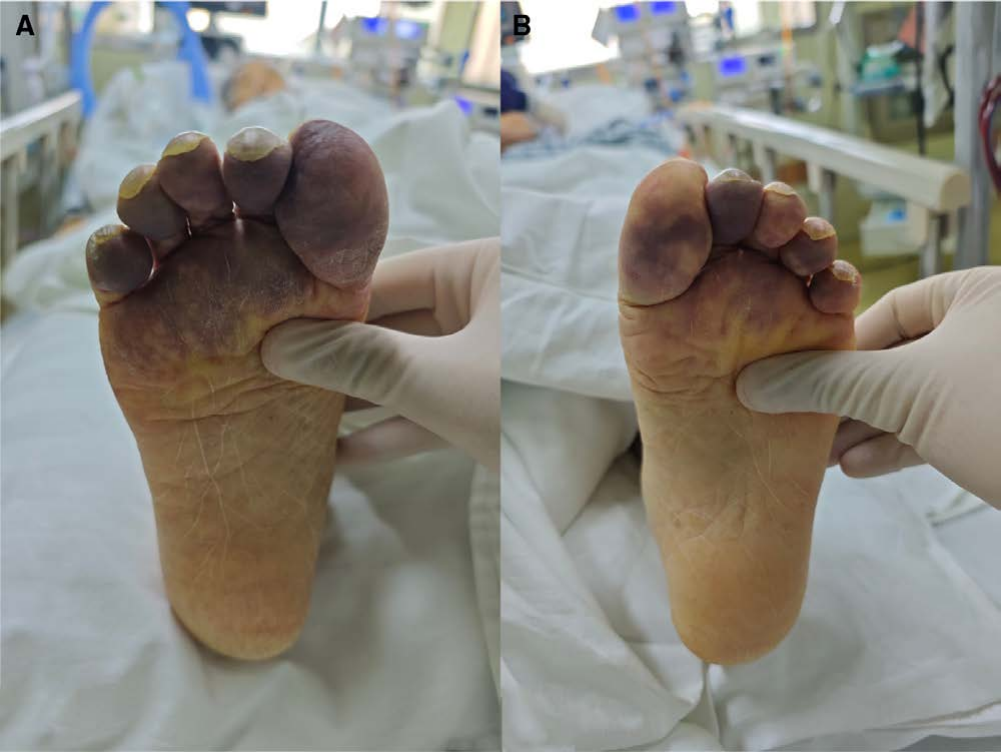
Clinical manifestations of peripheral circulatory impairment in both feet. Circulatory impairment in the toes and forefoot of the (A) right foot and (B) left foot.

A 66-year-old female was admitted to our hospital with a 9-year history of paroxysmal cough and wheezing, which had worsened over the preceding 5 days, and was accompanied by fever. Upon admission, she was diagnosed with pulmonary interstitial fibrosis and type I respiratory failure. She underwent bilateral lung lobe transplantation with venovenous extracorporeal membrane oxygenation (ECMO) and continuous renal replacement therapy. During the operation, she lost 1000 mL of blood and received transfusions of 800 mL of blood, 750 mL of plasma, and 250 mL of platelets. Postoperatively, she was transferred to the intensive care unit (ICU) with ECMO still in place. Empirical antibiotic therapy with imipenem-cilastatin sodium was initiated. The patient developed severe primary graft dysfunction after surgery and was treated with continuous renal replacement therapy and inhaled nitric oxide. Norepinephrine was administered via a microinfusion pump to maintain blood pressure, reaching a maximum dosage of 0.4 μg/kg/min. On the ninth day, after meeting the criteria for weaning, venovenous ECMO was discontinued. Due to the vasoconstrictive effects of norepinephrine on peripheral circulation, it was replaced with vasopressin at a maximum dose of 0.025 U/kg/h. Throughout her ICU stay, sputum, blood, pleural fluid, and throat swab cultures were monitored continuously. After 1 week, sputum cultures revealed carbapenem-resistant *Acinetobacter baumannii*, *Pseudomonas aeruginosa*, and methicillin-sensitive *Staphylococcus aureus*, prompting a switch to polymyxin E combined with ceftazidime-avibactam and eravacycline for infection control. On the 12th day, sputum cultures indicated the presence of *Burkholderia cepacia*, and the antibiotic regimen was maintained. On the 8th day, coagulation tests showed a prothrombin time of 14.4 seconds, a D-dimer level of 51,136 μg/L, a fibrinogen level of 0.87 g/L, and a platelet count of 66 × 10^9^/L. The diagnosis of DIC was confirmed based on an International Society on Thrombosis and Haemostasis score of 5. Figure 1 and Table S1, Supplemental Digital Content, https://links.lww.com/MD/P761 summarize the patients’ laboratory results during the ICU stay. On the 2nd day after the onset of DIC, the patient developed peripheral circulatory disturbances in the toes and forefoot (Fig. 2). Dorsalis pedis artery pulses were palpable, and Doppler ultrasonography revealed no vascular abnormalities. Based on the clinical manifestations, SPG was diagnosed.

**Figure 3.**
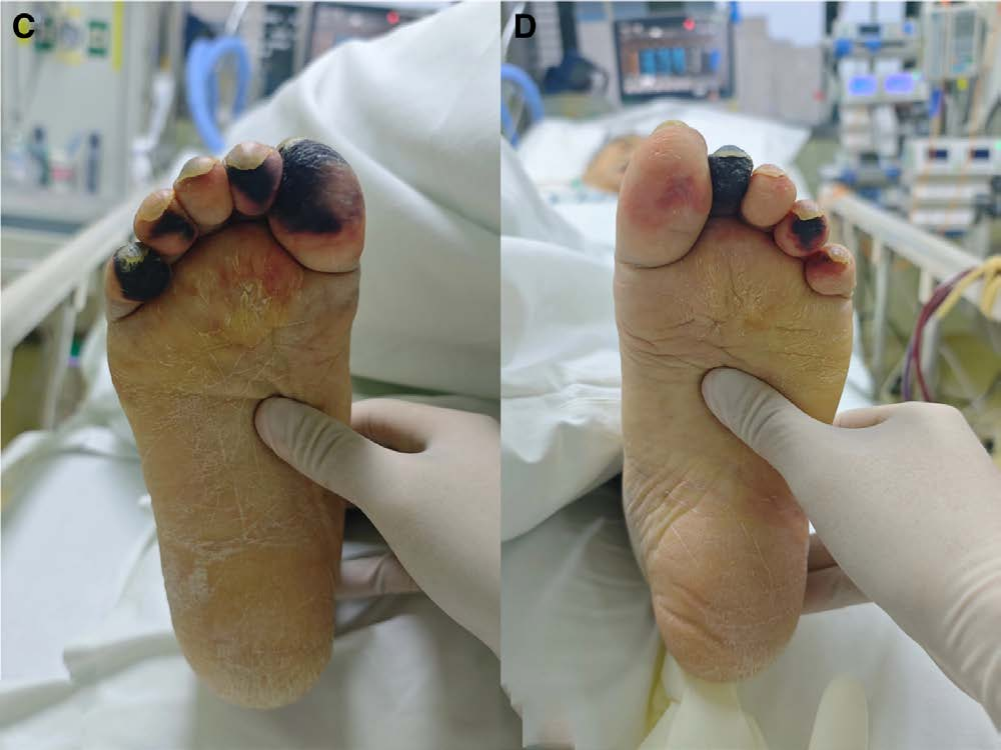
Clinical presentation of both feet on the 10th day of phentolamine wet compress. (C) Right foot and (D) left foot show significant improvement in the forefoot and some toes.

To manage the underlying condition, we promptly applied a warming blanket for temperature regulation, administered argatroban for anticoagulation, and treated microcirculatory disturbances using phentolamine wet compresses. A 10 mg dose of phentolamine was diluted in 20 mL of 0.9% sodium chloride for each application. Gauze was extended 5 to 10 mm beyond the affected area to ensure effective treatment at each site. The compresses were replaced every 20 to 30 minutes. By the second day of treatment, reversible skin color changes were observed, transitioning from cyanotic to ruddy. By the 10th day of treatment, the patient’s right big toe, right third toe, left third toe, and forefoot areas had nearly returned to normal. However, the right second toe and parts of the left toes developed irreversible circulatory disturbances, progressing to dry gangrene with clear demarcation lines (Fig. 3).

Unfortunately, on the 12th postoperative day, a computed tomography scan revealed a significant right-sided pneumothorax and mediastinal emphysema, and fiberoptic bronchoscopy identified a fistula at the anastomosis site of the right middle lobe. While enhancing nutritional support, a metal stent was placed via fiberoptic bronchoscopy, and closed thoracic drainage was performed on the right side to treat the pneumothorax. On the 18th postoperative day, liver function tests showed alanine aminotransferase 43 U/L, aspartate aminotransferase 74 U/L, γ-gamma-glutamyl transferase 35 U/L, cholinesterase 1977 U/L, and total bilirubin 157 μmol/L. Suspecting drug-induced liver injury, eravacycline was discontinued, the immunosuppressive was switched from tacrolimus to cyclosporine, and plasma exchange and bilirubin adsorption therapy were initiated. By the 27th postoperative day, there was no significant improvement in the anastomotic fistula. As the patient developed multiple organ failure, a decision was made in consultation with the family to discharge the patient voluntarily.

## 3. Discussion

SPG is a rare condition first described by Hutchinson in 1891 as symmetrical acral necrosis of the extremities.^[4]^ Since then, it has primarily been reported through case studies. Clinically, SPG presents with cyanosis, mottling, and symmetrical ischemia of the distal extremities, often progressing to gangrene.^[5]^ Patients with SPG generally have a poor prognosis, with reported mortality rates ranging from 40% to 90%.^[6]^ In this case, the patient developed septic shock and DIC following lung transplantation, which was further complicated by SPG.

However, the underlying pathophysiological mechanisms of SPG remain poorly understood. It is most commonly associated with shock, DIC, and the depletion of natural anticoagulants such as protein C and antithrombin.^[7]^ Shock is frequently linked to the use of vasoactive drugs. Low-dose dopamine (2–5 μg/kg/min) causes vasodilation in coronary, renal, and mesenteric vessels, whereas high doses (20–50 μg/kg/min) activate α-receptors and induce vasoconstriction.^[8]^ Norepinephrine, a first-line agent for managing septic shock, may exert an even more pronounced effect on microcirculation. The combined effects of hypotension and vasopressor administration compromise peripheral blood flow, resulting in ischemia, necrosis, and gangrene. However, the link between vasoactive agents and SPG remains controversial, as drug-induced peripheral ischemia typically appears within hours of administration, rather than the 36 to 48 hours reported in most SPG cases.^[7]^ Alternatively, hepatic ischemia, also known as shock liver, may better explain this delayed pathophysiological response.^[6]^ Approximately 85% of SPG cases are reportedly associated with DIC, suggesting it may represent a final common pathway in the disease’s pathogenesis. Consequently, patients with septic shock and DIC are more susceptible to peripheral microthrombosis, ultimately resulting in ischemia, necrosis, and gangrene.^[9]^

In SPG, the fingers and toes are most commonly affected, while involvement of the nose, earlobes, and scrotum is less frequent.^[10]^ In this case, only the patient’s nasal tip, ear tips, and trunk were unaffected, and circulatory disturbances were limited to the lower limbs. The patient initially presented with cold, pale feet, preserved arterial pulses, and no vascular abnormalities on Doppler ultrasonography. After 2 days, significant peripheral ischemic changes were observed, including cyanosis of the toes and forefoot. Some toes progressed to dry gangrene within 10 days, accompanied by partial nail loss, which is consistent with previous reports.^[11,12]^ The demarcation line of necrosis usually develops within 2 weeks, guiding decisions regarding the extent of amputation.^[13]^

Currently, no standardized, evidence-based guidelines exist for the treatment of SPG. Most treatment strategies have been described in individual case reports. Described treatments for SPG include sympathetic nerve blocks, topical or injected nitroglycerin, intravenous prostaglandins, and subcutaneous botulinum toxin injections.^[14–16]^ Phentolamine, an α-adrenergic blocker, induces vasodilation by relaxing vascular smooth muscle and inhibiting the binding of norepinephrine and epinephrine to their receptors. Numerous reports have documented successful treatment of skin necrosis caused by dopamine or norepinephrine extravasation and ischemia from accidental epinephrine injection. Phentolamine is also widely used to manage vasospastic conditions such as Raynaud syndrome and thromboangiitis obliterans.^[3,17,18]^ However, there are a few reports of its use in treating peripheral ischemia in SPG secondary to septic shock and DIC. On the 2nd day of circulatory disturbance, we opted for phentolamine wet compresses instead of local injections, as reported in previous cases. This approach is clinically feasible for medical staff and reduces the risk of infection and injection-related side effects. By the 8th day of treatment, significant improvement was observed in ischemia of the forefoot and some toes, although some areas progressed to irreversible dry gangrene. Currently, the only definitive treatment for SPG is amputation, with microsurgical free-flap transplantation being the optimal surgical choice.^[10]^

In conclusion, correcting risk factors and early recognition of SPG are more crucial than interventions initiated after onset. For patients with shock, DIC, or acute ischemic hepatitis, minimizing the dose of vasoactive drugs, especially peak doses, while ensuring circulatory stability,^[19]^ and actively treating the primary disease are essential. Peripheral perfusion should be assessed using capillary refill time, peripheral-to-core temperature difference, and peripheral perfusion index.^[20]^ We believe that if preventive treatment had been initiated during the early or reversible phase of peripheral ischemia, the patient’s lower limb ischemic complications could have been better managed. Additionally, local wet compresses, which are typically applied to small areas in SPG treatments, ensure safety. No adverse effects related to phentolamine were observed. Although clinical trials on phentolamine for SPG are still lacking, our findings are promising.

## Author contributions

**Conceptualization:** Yichao Zhu, Haohao Qian, Hongyang Xu.

**Data curation:** Yichao Zhu.

**Formal analysis:** Yichao Zhu.

**Funding acquisition:** Hongyang Xu.

**Investigation:** Yichao Zhu.

**Methodology:** Yichao Zhu.

**Project administration:** Hongyang Xu.

**Supervision:** Tao Zhou, Hongyang Xu.

**Visualization:** Yichao Zhu, Haohao Qian.

**Writing – original draft:** Yichao Zhu.

**Writing – review & editing:** Yichao Zhu, Tao Zhou, Hongyang Xu.

## Supplementary Material


